# Exploring the Role of Nutraceuticals in Major Depressive Disorder (MDD): Rationale, State of the Art and Future Prospects

**DOI:** 10.3390/ph14080821

**Published:** 2021-08-21

**Authors:** Miguel A. Alvarez-Mon, Miguel A. Ortega, Cielo García-Montero, Oscar Fraile-Martinez, Jorge Monserrat, Guillermo Lahera, Fernando Mora, Alberto Rodriguez-Quiroga, Sonia Fernandez-Rojo, Javier Quintero, Melchor Alvarez-Mon

**Affiliations:** 1 Department of Medicine and Medical Specialities, University of Alcala, 28801 Alcala de Henares, Spain; maalvarezdemon@icloud.com (M.A.A.-M.); cielo.gmontero@gmail.com (C.G.-M.); oscarfra.7@hotmail.com (O.F.-M.); jorge.monserrat@uah.es (J.M.); guillermo.lahera@gmail.com (G.L.); mademons@gmail.com (M.A.-M.); 2 Ramón y Cajal Institute of Sanitary Research (IRYCIS), 28034 Madrid, Spain; 3 Department of Psychiatry and Mental Health, Hospital Universitario Infanta Leonor, 28031 Madrid, Spain; fernando.mora@salud.madrid.org (F.M.); arquiroga@salud.madrid.org (A.R.-Q.); sfernandezr@salud.madrid.org (S.F.-R.); fjquinterog@salud.madrid.org (J.Q.); 4 Cancer Registry and Pathology Department, Hospital Universitario Principe de Asturias, 28806 Alcalá de Henares, Spain; 5 Psychiatry Service, Center for Biomedical Research in the Mental Health Network, University Hospital Príncipe de Asturias, 28806 Alcalá de Henares, Spain; 6 Department of Legal Medicine and Psychiatry, Complutense University, 28040 Madrid, Spain; 7 Immune System Diseases-Rheumatology, Oncology Service an Internal Medicine, University Hospital Príncipe de Asturias, (CIBEREHD), 28806 Alcalá de Henares, Spain

**Keywords:** major depressive disorder (MDD), nutraceuticals, omega 3 fatty acids, S-Adenosyl-methionine (SAMe), vitamin D, methylfolate, pre/probiotics, micronutrients, bioactive compounds

## Abstract

Major depressive disorder (MDD) is a complex and common disorder, with many factors involved in its onset and development. The clinical management of this condition is frequently based on the use of some pharmacological antidepressant agents, together with psychotherapy and other alternatives in most severe cases. However, an important percentage of depressed patients fail to respond to the use of conventional therapies. This has created the urgency of finding novel approaches to help in the clinical management of those individuals. Nutraceuticals are natural compounds contained in food with proven benefits either in health promotion or disease prevention and therapy. A growing interest and economical sources are being placed in the development and understanding of multiple nutraceutical products. Here, we summarize some of the most relevant nutraceutical agents evaluated in preclinical and clinical models of depression. In addition, we will also explore less frequent but interest nutraceutical products which are starting to be tested, also evaluating future roads to cover in order to maximize the benefits of nutraceuticals in MDD.

## 1. Introduction

Major depressive disorder (MDD), also known as clinical depression or major depression, is a debilitating condition characterized by altered mood, impaired cognitive functions, anhedonia (the inability to feel pleasure), and vegetative disorders like insomnia, fatigue or anorexia [1]. It is estimated that MDD affects over 350 million people worldwide, representing about 4.7% of the global population with approximately a 3% incidence per year [2]. Following the Global Burden of Diseases, Injuries, and Risk Factors Study 2017 (GBD 2017), MDD is the third highest cause of disability in the world, just after headache disorders and low-back pain [3]; however, the World Health Organization (WHO) projections estimate that MDD will be placed as the leading cause of disability by 2030 [4]. Epidemiologically, females are frequently more affected than men, in which a heavier role of genetics is suspected [5]. Age, ethnic, hormonal and sociocultural factors are also important determinants to suffer from MDD, as well as the presence of certain comorbidities and chronic maladies [6].

MDD could be manifested in several forms depending on each individual, entailing a plethora of possible clinical symptoms. According to the *Diagnostic and Statistical Manual of Mental Disorders* (DSM-5), to be diagnosed with MDD, a patient should present at least depressed mood or anhedonia (both considered main criteria) and ≥4 secondary symptoms, divided into somatic items (e.g., sleep disturbances, appetite disorders or fatigue) versus non-somatic or affective factors (like feelings of worthlessness and suicidal thoughts). Importantly, these manifestations should appear during a period of ≥2 weeks [7,8,9]. In order to establish the severity of the MDD, the Hamilton Depression Rating Scale (HAM-D) is globally used, with specific punctuation marks for each clinical parameter [10]. Thus, a range from 0–7 is diagnosed as no depression; 8–16 is corresponded to mild depression; 17–23 as moderate depression; and ≥24 is considered severe depression [11]. Regarding the DSM-5, anhedonia and non-somatic symptoms are usually associated with severe depression while depressive mood and somatic items are commonly indicators of moderate depression [12]. Clinical management of MDD could be distinct according to the severity. For mild depression, physical activity or non-medical therapies could be recommended. Moderate cases might need antidepressant monotherapy, psychotherapy or a combination of both. For the severe presentations, the combined use of an antidepressant and an antipsychotic, electroconvulsive therapy or the combination of an antidepressant plus psychotherapy can be required [13]. On the other hand, patients with severe cases had worse clinical outcomes despite receiving greater attention and treatments [14], and approximately 30% of patients with MDD suffer from therapy resistance, thereby limiting their clinical management [15].

On the other hand, the clinical management of this condition entails an elevated economic burden, including not only direct, but also indirect costs, due to decreased productivity or work absence [16]. Only in the United States, the estimated direct and indirect costs are about $210 billion per year [17]. It is of note that, the economic hit of MDD is significantly high independently from the age, with the greatest added costs in adolescents and younger patients [18]. Besides, either treatment-resistant depression or severe cases of MDD are associated with high direct and indirect costs, respectively [19], thus concluding the importance of preventing the onset of depression in younger people and considering severe presentations of MDD and treatment-resistant cases. MDD is also related to a poor quality of life (QOL) of the affected patients. Here, it should be highlighted the social dysfunction of these individuals, and particularly in the impaired attachment and affiliation, social communication as well as altered perception and understanding of others [20]. In addition, it is unclear if proper medical care improves the QOL of patients with MDD. Ishak et al. [21] show that despite 30% of patients after treatment reported “normal” QOL parameters in comparison to the 3% before treatment, >50% of patients exerted severely impaired QOL measures, and even non-remitters had reduced QOL after one year follow-up. Thus, a multidisciplinary approach is essential to assure a proper management of these patients, counting on an adequate balance of medical and non-medical treatments like psychological therapy [22], physical activity [23,24] or nutritional interventions [25]. However, more efforts are needed to understand the complex nature of MDD, and further research must be established to improve the clinical management of these patients, with special focus on their QOL.

## 2. Pathophysiology of MDD

MDD is a complex multifactorial disease with many biological and non-biological factors involved. The origin of MDD is complex. MDD might be understood as a disease of modernity. The mismatch of our evolutive design, leading to an altered mental and physical well-being is one of the most plausible explanation for the increased prevalence of depression in the world. In addition, declining social capital, feelings of loneliness and greater inequity are also major contributors for the depressiogenic environment [26,27,28].

The heritability of the disease is estimated about 30 to 40% as demonstrated in previous studies conducted in twins [29]. Despite multiple genes have been associated with MDD, the evidence has failed to find consistent proof of the role of single gene variants in the pathophysiology of MDD, concluding that (a) the impact of genetics is lower than expected and/or (b) that MDD is a heterogeneous malady and the genetic burden could be quite different among individuals [30,31,32,33]. Regarding environmental factors, lifestyle and, prominently, the exposure to stressful events during early life (including prenatal and perinatal periods) are likely related to the onset and development of MDD [34]. In addition, psychological (personality, coping style, thoughts) and social factors (family and social relationships, employment, life events) are also major contributors of suffering from MDD [35]. All these mechanisms, such as genetic, environmental and psychosocial factors, promote a set of changes in the human brain, which leads to the development of MDD. At the core of these interactions is epigenetics. Epigenetics refers to those changes occurred in the gene expression but without affecting the DNA sequence. The most important epigenetic mechanisms include DNA methylation, histone modifications and non-coding RNAs (e.g., micro RNAs (miRNAs)) [36]. In patients with MDD, several changes in all these systems have been described, having been proposed not only as a potential therapeutic target but also as valuable predictive and diagnostic biomarkers [37,38]. Notwithstanding that there is enough evidence to support the central role of epigenetics in the pathogenesis of MDD, further studies are needed in this field to find effective opportunities in the epigenetic reprogramming of this condition [39].

Traditionally, the pathophysiology of MDD has been associated with an abnormal functioning of various neurotransmitters, especially serotonin, dopamine or norepinephrine. This is designed as the monoamine hypothesis, and many of the antidepressants currently used are based on this theory [40]. In this context, serotonin (5-HT) is the most studied neurotransmitter in MDD. Different alterations could be reported in all the serotoninergic pathway, including changes in the 5-HT receptors and the 5-HT membrane transporter protein (SERT) [41]. In addition, impaired serotonin synthesis by the metabolite tryptophan could also lead to MDD, increasing the production of some potentially neurotoxic compounds like quinolinic acid [42]. Norepinephrine is another neurotransmitter of note in the pathogenesis of MDD, taking part in the emotional and cognitive dysfunctions in these patients [43]. Dopamine circuit also play a central role in MDD, being intimately associated with patient’s anhedonia [44]. However, despite this, there is no denying the fact that monoamines are implicated in the pathophysiology of MDD. It is difficult to establish if these alterations are cause, consequence or an adaptation to this condition. Moreover, this theory fails to explain why there are some patients with late or no response to the treatment received, thereby concluding that more complex mechanisms might be working in the pathophysiology of the disease [45,46]. For instance, a growing amount of evidence claim an altered functioning in the inhibitory gamma-aminobutyric acid (GABA) interneurons and excitatory glutamate neurons, with potential therapeutic opportunities [47]. Furthermore, multiple studies are focusing on the relevance of the circadian rhythms and its main mediator, melatonin in the onset and development of the disease, although its therapeutic translation is still unclear [48,49].

Glucocorticoids like cortisol (the stress hormone) play a central role in the changes occurred in the brain of patients with MDD and its pathogenesis. Glucocorticoids are released by the adrenal glands in response to the adrenocorticotropic hormone (ACTH) secreted by the pituitary, just after the production of corticotropin releasing hormone (CRH) by the paraventricular nucleus (PVN) localized in the hypothalamus. This system is known as the hypothalamic–pituitary–adrenal (HPA) axis, and it is activated under stress conditions [50]. The acute activation of this axis was associated with survival and fight-or-flight responses, exerting a plethora of biological effects. However, chronic exposure to glucocorticoids negatively impacts the whole functioning of the organism. The brain is the hardest-hit structure affected by HPA axis dysfunction. Indeed, neuroimaging studies have shown multiple critical brain regions which appear to be affected in patients with MDD, particularly in cortical and subcortical limbic areas, basal ganglia and brain stem [51]. All these areas may present important modifications in their volume, with augmented neuronal death and decreased neurogenesis as well as reported changes in the brain network, affecting glial and neuronal plasticity [52]. In this line, it seems that the altered activity at some neurotrophins like brain-derived neurotrophic factor (BDNF) might contribute to the decreased brain plasticity in patients with MDD, and different studies have tried to evaluate its use as biomarker in clinical practice, although available evidence is still controversial [53,54,55].

To fully understand the causative agents of MDD, the role of the immune system should also be mentioned. A plethora of immune cytokines have been found to be implicated in the pathophysiology of depression, including interleukins (IL)-1β, IL-2, IL-4, IL-6, IL-8, IL-10 interferon gamma (IFN-γ), C-reactive protein (CRP), tumor necrosis factor α (TNFα) and the chemokine monocyte chemoattractant protein-1 (MCP-1) [56,57]. The exacerbated cytokine release observed in depressed patients may pass the brain–blood barrier, activating the glial cells and leading to a neuroinflammatory process; therefore, this contributes to the previously described damage in the brain [58,59]. Furthermore, an excessive stress negatively impacts the immune system, which in turn affects the HPA axis. Both factors lead to neurological impairments in the brain, causing changes in mood and behavior. These changes in the brain are accompanied with more inflammation and stress in a prolonged vicious cycle. The link between psychological, neurological, endocrine and immune function is collected under the term “psychoneuroimmunoendocrinology”, and it is the most important target to address in MDD [60].

Oxidative stress (OS) is another pathophysiological mechanism which may be involved in MDD. This condition appears following a reduction in antioxidant levels and an augmentation in the free radicals, which are mainly reactive oxygen/nitrogen species. The brain is highly susceptible to the oxidative damage, and previous studies have mentioned the implication of OS in neurodegenerative and psychiatric disorders, including MDD [61,62,63]. In addition, compelling evidence shows patients with MDD present with substantial variations in different metabolic factors including altered glucose, lipids and albumin profile, as well as abnormal leptin, ghrelin and insulin activity [64]. Last but not least, recent insights into the systemic alterations of MDD has focused their attention on the gut microbiota. Gut and brain are two structures connected at multiple levels, and the set of microorganisms inhabiting the gut and their products are essential in this bidirectional communication, conforming the MGB axis [65,66]. Depressed patients have significant changes in the gut microbiota (dysbiosis) in comparison to healthy patient, leading to a proinflammatory status and neuroinflammation, enhancing the HPA axis dysfunction and stress sensitivity in the brain and disrupting the gut–brain communication through the vagus nerve, hence contributing to the pathogenesis of MDD [67]. An altered immune status described in MDD is responsible for an enhanced bacterial translocation in the bloodstream, aggravating the systemic damage in depressed patients [68,69]. Moreover, accumulating evidence have described the central role of gut microbiota either in the production or degradation of multiple neurotransmitters, including serotonin, norepinephrine, dopamine or GABA [70], defining the gut microbiota as a critical modulator of brain activity.

Therefore, as described here, there are a plethora of causative mechanisms involved in the pathogenesis of MDD, including psychoneuroimmunoendocrinological alterations, metabolic factors, increased oxidative stress and altered microbiota–gut–brain axis. Thus, an integrative approach of MDD is critical to address this global and growing concern, including medical care, proper nutrition, physical activity and psychotherapy. In this sense, the present review examines the role of nutraceuticals as a potential therapeutic agent in patients with MDD, exploring the rationale of their use; current preclinical and clinical trials; and future research paths are discussed.

## 3. Nutraceuticals: Definition and Context

Nutraceuticals are foods, or part of them which are essential to prevent the appearance of different pathological conditions, also working as a potential adjunctive therapy in the management of established diseases [71]. The term was first introduced in 1989 when Dr Stephen DeFelice joined “nutrition” and “pharmaceutical” concepts. Nevertheless, marketing campaigns have overused the term in the food industry and pharmacy for supplements and there are regulatory definitions in different countries [72]. Indeed, there is no universal definition yet but, in most countries, they refer to dietary supplements. However, some authors differ between the terms nutraceutical, functional foods and dietary supplements. Functional foods refer to “scientific strategies” when cooking or preparing a concrete food, in order to maximize their nutritional benefits, playing a key role in the health maintenance. However, when this food aids in the prevention and/or treatment of certain diseases/disorders, they are considered nutraceuticals. Nutraceuticals could also be isolated components. Nonetheless, they differ from dietary supplements as their function is not only supplement the diet, but also therapeutical, and that they may also serve for use as a conventional food or as the sole item of meal or diet [73]. Other authors claimed the urgency of finding an adequate definition of nutraceuticals or its substitution by other more precise and adequate term [74]. In this review, we will address the concept in terms of “natural nutrient drug” to focus on the properties that certain components naturally present in food, purified or optimized may offer besides its interactions with pathophysiological mechanisms in MDD. Matching with this definition, in the 1990s, DeFelice had already claimed that these products designed by industry must demonstrate their clinical benefits with evidence to grow in success [75]. Currently, the development of safe nutraceuticals with enhanced delivery properties also owes their success to nanotechnology and biotechnology with liposomes, nanoparticles and dendrimers, among others [72], as well as the use of “food matrix”, an integrative set of components that may potentiate the bioavailability and benefits from nutraceuticals [76].

The idea of food with therapeutical effects is not something new though, as Hippocrates said, “Let food be thy medicine”. This added to the public interest in therapeutic agents of natural origin and, in contrast to the adverse effects of many chemically synthesized drugs have provided nutraceuticals major acceptance when pharmaceutical treatment fails [77]. On the other hand, the costs normally are much lower for nutraceuticals, and they do not require medical prescription, making their access even easier. Hence, nutraceuticals have gained fame from the beginning of 21st century greatly due to their cost, easy access, tolerability and safety [78]. Moreover, the short regulation by authorities allows their easy access and buying over-the-counter, different from pharmaceuticals [79]. However, sometimes the lack of medical control in some patients can cause problem, especially when interaction with medication can be damaging, or even if the individual does not make the proper use of it with the incorrect doses and without telling their physician [77]. It is of note that the global market of nutraceuticals is incredibly huge, accounting for $379.061 billion in 2017 and it is expected to almost duplicate its value to USD 734.601 billion by 2026 [80]. It means that there is a growing interest in the development and applications of nutraceuticals in different circumstances. 

As will be reviewed, there are many pilot studies and animal models demonstrating a plethora of effects in the brain, being designed by some experts as neuro-nutraceuticals [81]. However, more blind randomized controlled trials and nutraceutical-therapeutic drugs studies are still needed in order to assure safety, efficacy and cost-effectiveness. In the area of depression, the undesirable response to antidepressants has led to the proposal of nutraceutical adjuvants. Moreover, many patients with MDD are malnourished, with either reported deficiencies in some nutrients or more prominently an excessive body mass index (BMI) [82]. Several clinical trials have been conducted over the last decade, remarking on the adjunctive use of omega-3, vitamin D, S-adenosylmethionine (SAMe) and methylfolate [83]. Moreover, there are preclinical and clinical studies conducted regarding the role of other nutraceuticals including micronutrients, prebiotics, probiotics, creatine, aminoacids or plant-derived bioactive compounds. In this work we will summarize current knowledge of the applications of these nutraceuticals in MDD, in order to create an integrative perspective of the possible uses and translational opportunities in this condition

## 4. Nutraceuticals in MDD

### 4.1. Omega 3 Denosyl-Methionine

The epidemiology alleges that high intake of the major source of ω-3 polyunsaturated fatty acids (ω-3 PUFAs), fish, is associated with lower prevalence of MDD. In fact, depression is a less common disorder in those countries with huge fish consumption, so the primary hypothesis was that fish oil is a good ingredient to prevent or treat MDD. In clinical trials, MDD patients that are under treatment show better outcomes when fish oil is taken compared to placebo. Interestingly, Burhani and Rasenick [84] reviewed that some sites of action of PUFAs at the cell membrane are G-proteins within lipid rafts. At this site of cell, every change in its proteins’ localization can be translated into alterations in neurotransmitters signaling. These proteins are also targets for antidepressants, so the status of lipid rafts may play a prominent role in the effectiveness of the treatment. On the other hand, when located in lipid rafts, Gα subunits cannot couple to adenylyl cyclases (ACs) to synthesize cyclic adenosine monophosphate (cAMP), but PUFAs and antidepressants have the ability to translocate Gα subunits out of the lipid rafts, making them accessible by ACs to form complexes. In a meta-analysis and meta-regression about ω-3 PUFAs supplementation in MDD patients, they observed that the major clinical benefit is given when, more specifically, eicosapentaenoic acid (EPA) is taken in higher dose (ω-3 PUFAs with EPA ≥ 60% at a dosage of ≤1 g/d) and this benefit is not observed for docosahexaenoic acid (DHA) [85,86]. Another meta-analysis showed the effective dose response for EPA+DHA supplements in clinical trials was slightly different, where EPA ≥ 60% of total the dose range was 200 to 2200 mg/d of EPA more than DHA [87].

The underlying mechanism is contrasted in animal and in vitro models, where EPA seems to stimulate the expression of myelin proteolipid proteins (PLP), the main myelin proteins in the central nervous system (CNS), via cAMP-mediated pathways [88,89]. These results corroborated the neuroprotective effects of ω-3 PUFAs against demyelination in diseases like multiple sclerosis [90]. Although these mechanisms are not entirely understood yet for MDD in the context of etiology, these results correlate with the decrease of myelin in the axons of callosal splenium in in vivo and postmortem brains from depressed subjects, an observed characteristic aspect for this pathology and not for others like schizophrenia [91,92]. In another study with magnetic resonance, they found disturbances in myelin integrity in the fornix [93], which is key for memory, another impaired feature in MDD. In this context, ω-3 PUFAs have been associated with a delay in cognitive decline, DHA is especially associated with memory maintenance. Its higher proportion in hippocampal due to a high-DHA diet is correlated to higher retention ability in mice [94]. In animal models, recently it has been observed that both EPA and DHA are critical to keep at a natural ratio like that in fish oil. An excess of EPA may provoke learning and memory impairment by GABAergic transmission enhancement, but DHA is able to prevent this damage [95]. Thus, the combination of both is key when considering supplementation in humans. Other rat studies have also noticed that supplementation with ω-3 PUFAs, in general, refines dendritic architecture and improves spatial memory [96].

Kalkman et al. [97] checked that EPA also denotes more antidepressant effects because its lipid metabolites have endocannabinoid behavior now that they find affinity in cannabinoid receptor-2 (CB2). These derived metabolites imply effects on immunomodulation, neuroinflammation, food intake and mood. Endogenous endocannabinoids are synthesized from ω-6 and ω-3 PUFAs, but anti-inflammatory effects are observed with the second ones. Little is still known about the molecular mechanisms and targets, but their conjugation with neurotransmitters 5-HT and dopamine has been associated with pain relief and inflammation lessening [98]. Neuroinflammation studies demonstrated that these ω-3 endocannabinoids are metabolized mainly by cytochrome P450 (CYP) generating epoxides with the ability to lessen proinflammatory IL-6 while boosting anti-inflammatory IL-10 production by the activation of CB2 receptor [99].

The interaction of adjuvant EPA with certain antidepressants is suggested to improve the response in MDD patients. In some in vitro studies, the addition of ω-3 PUFAs to escitalopram treatment (selective 5-HT reuptake inhibitors) has shown to increase ACs activity and BDNF expression in human lymphoblast cell lines from depressed patients, avoiding cAMP accumulation [100].

All data that has been recorded through scientific evidence related to ω-3 PUFAs in the treatment of depression at least did not show associated side effects, what signifies an advantage for those patients that do not respond to pharmaceutical treatment or present side effects dealing with the same one [101]. Thus, ω-3 PUFAs with EPA ≥ 60% + DHA are definitively nutrients with pleiotropic effects including the anti-inflammatory action, the neuroprotective effect with the signaling membrane proteins modification and the endocannabinoid effect; all of them contribute to the antidepressant action but these mechanisms still need to be more elucidated.

Obviously, ω-3 PUFAs are not going to show prompt clinical benefits per se if the patient is not previously well nourished, just like any pharmaceutical or dietary supplement is not going to be successful unless the individual is monitored in a period of few months [102]. Moreover, more beneficial effects have been seen in patients with longer treatment duration and with mild to moderate depression and with no depression, but more quality evidence is still missing [103].

### 4.2. Vitamin D

Fat-soluble vitamin D consist in different forms of a steroid hormone. Vitamin D_3_ (also called 1, 25-dihydroxycholecalciferol or calcitriol) is produced in the skin when ultraviolet radiation from sun exposure obtains 7-dehydrocholesterol [104]. The main source of this nutraceutical in humans is through solar exposure. D_3_ which is more efficient, and is also found in oily fish, cod liver or milk, while D_2_ (ergocalciferol) is found in some vegetables.

Vitamin D deficiency has been studied in relation to depression to establish if there is risk or causality for the physiopathology. The current evidence cannot assure a clear link; however, clinical data has demonstrated that hypovitaminosis D in MDD patients has an association with the symptoms. The lack of this micronutrient is not known to be a cause or a consequence of the disorder [105]. At the Clinical Trials platform, there is still a small number of trials registered about the use of vitamin D in depressive patients.

On the other hand, what is perfectly known is that this micronutrient is crucial for the correct functioning of gut microbiome and gut-associated lymphoid tissue (GALT). A deserving reason for the study of vitamin D deficiency in MDD is its role in signaling pathways for intestinal innate immunity and gut microbiota maintenance.

MDD usually accompanies the beginning of inflammatory bowel disease (IBD), in addition to anxiety, alexithymia and other psychological impairments [106]. On the one hand, the gut and brain are bidirectionally connected by the autonomic nervous system, HPA axis and nerves within the gastrointestinal tract; all of these contribute to influencing intestinal activities with their echo not only in GALT immunity but also in mood, cognition and mental health [107]. On the other hand, an impairment in the MGB axis has been included in the network of factors involved in the pathogenesis of depression. Thus, changes in microbiota composition and dynamics have been observed in MDD patients: composition alterations increase gut permeability, promotes inflammation, changes quantity of BDNF and alters the release of monoamine neurotransmitters [108]. Environmental factors like diet, stress or antibiotics and other pharmaceuticals, make direct effects on this MGB axis, leading to these kinds of alterations in health and disease [109]. Targeting gut microbiota has been suggested for the last decade, i.e., keeping or restoring the eubiosis status of microbiota as therapy or prevention for mental disorders [110]. In this context, recent evidence alleges that vitamin D is crucial for gut homeostasis, in fact, the vitamin metabolites act synergistically with microbiota metabolites as part of its role in the immunoregulation [111]. The main objective of vitamin D in GALT is to guarantee there is a proper concentration of antimicrobial peptides in the mucus layer to maintain tight junctions in the epithelial barrier [112]. It is also crucial for calcium absorption; the deficiency reduces it entailing gut stasis and increasing permeability, allowing the transference of lipopolysaccharides into the blood [113].

Shelving the dilemma of cause or consequence, being an additional factor in the physiopathology, it is of note their direct and indirect effects on sleep regulation and mood. Findings seem inconclusive but there has been described the serotonergic pathway in which vitamin D and UVB exposure modulate serotonin and melatonin. Huiberts and Smolders [114] scanned the literature related to vitamin D influence on serotonin and melatonin and proposed to include mood and sleep parameters in future studies in order to check the action mechanism of vitamin D on mood by quality of sleeping. It is of note that vitamin D and melatonin are inversely related by sun exposure and both molecules may share common mechanisms [115] that could be relevant for MDD.

The association with risk of sleep disorders is found in the scientific literature of vitamin D deficiency accompanied by shorter sleep duration or poorer sleep quality [116]. In a randomized eight-week follow-up trial, 89 subjects (44 in intervention group and 45 in placebo group) were examined in terms of sleep quality. The intervention group received 50,000 IU/fortnight and the results showed significant differences compared to the placebo group. The study concluded that supplementation could improve sleep quality and sleep duration in people with sleep disorders [117].

What is known so far is that brain serotonin is synthesized from tryptophan and this pathway is mediated by the vitamin D hormone (calcitriol), which activates the transcription of the serotonin-synthesizing gene tryptophan hydroxylase 2 (TPH2) in the brain. For this reason, a practical adjuvant for MDD could be vitamin D in combination with tryptophan as supplementation. About these key nutraceuticals mentioned above, improper levels of vitamin D, EPA or DHA can heavily disturb serotonin activation and function [118,119]. These data are contrasted in animal models that explain the link of vitamin D deficiency to neuropsychiatric disorders. Optimal concentrations of vitamin D can enhance serotonin synthesis and even is able to mimic MAO inhibitors and virtually increasing serotonin in the CNS [120].

Further research is required in matters of supplementation with vitamin D, timing, dosage of supplement and studying each case individually. In a nine-week follow-up big trial with 940 adolescent girls, vitamin D_3_ was administered at a dose of 50,000 IU/week [121]. The results found a significant reduction on depression score, measured by the Beck Depression Inventory II [122], implying that high dose of vitamin D supplementation may relieve depressive symptoms [121]. Although there are different results from observational studies, many of them found vitamin D deficiency as a possible risk factor for late-life depression [123].

There is no doubt that vitamin D is key for healthy brain function. In spite of lacking evidence about its implications in MDD, the mechanisms in which it intervenes are disturbed in MDD, demonstrating its relevance once again for invest in more research of this potential nutraceutical in combination with others above mentioned like ω-3 PUFAs [118].

### 4.3. S-Adenosyl Methionine

SAMe is a metabolic product synthesized during the cycle of methionine (met) through the enzyme methionine-adenosyltransferase (MAT2A). SAMe is considered the universal methyl donor in the living organisms. After transferring its methyl group, SAMe derivates to S-Adenosylhomocysteine (SAH), leading to Homocysteine (Hcys) formation when hydrolyzed by the enzyme SAH hydrolase. Then, Homocysteine methyltransferase (HMT) remethylates HCys to form met, thereby completing the met cycle [124]. The last reaction links met and folate cycle, both belonging to the “one carbon cycle”, which is a metabolic network that integrates nutrient status from the environment to fulfill a plethora of biological functions [125]. In the case of SAMe, it is considered a master epigenetic regulator, with several recognized functions in the central nervous system, mainly through cellular transmethylation pathway, inducing methylation of the DNA, histones, protein phosphatase 2A and various catecholamine moieties [126].

SAMe was first discovered by Cantoni in 1952. Since its introduction in the late 1970s in Europe and the late 1990s in the USA, the use of SAMe has been explored in a wide variety of medical conditions including bone and liver diseases, fibromyalgia, neurodegenerative disorders and, more prominently, MDD [127]. Different changes have been reported in SAMe and one carbon cycle, hence contributing with the pathophysiology of the disease [128]. For example, lower levels of MAT2A and SAMe may negatively affect the regulation of some critical components of monoaminergic neurotransmitters either by direct mechanisms (modulating their catabolic enzymes, transporters and receptors) and indirect (influencing the synthesis of tetrahydrobiopterin (BH4) cofactor, a critical component involved in monoamine synthesis) [127]. Moreover, previous studies have found substantial alterations on the SAH/SAMe ratio in patients with MDD, together with an altered methylation index [129]. This abnormal methylation status could influence adversely certain critical targets like BDNF; therefore, playing a relevant role in the onset and progression of MDD [130]. In addition, SAMe may favorably operate through further mechanisms, participating in the downregulation of several inflammatory mediators and gut dysbiosis [131]. Thereby, the administration of SAMe has been proposed as a potential therapeutic approach in patients with MDD. However, it is important to understand to contextualize the role of this nutraceutical in the clinical management of MDD.

Most of the trials, systematic reviews and meta-analysis report similar conclusions regarding SAMe administration in patients with MDD. Notwithstanding that the use of SAMe is generally safe, well-tolerated and it may exhibit some improvements in patients with MDD, the quality of evidence of this nutraceutical is not high enough and further efforts in this field are required [132,133,134,135,136]. Indeed, there are certain studies with no benefits observed from SAMe supplementation, particularly, when compared to classical standard treatments [137,138]. Likewise, the benefits of SAMe appears to be sex-dependent, with men being more sensitive than women, although the precise causes of these differences have not been fully elucidated [139]. It seems more appropriate that SAMe be used neither as monotherapy nor substitutive approach but as an adjunctive therapy. For instance, a clinical trial demonstrated the advantages from using SAMe versus placebo as adjuvant of serotonin reuptake inhibitor (SRI) in non-responder patients [140]. Their results show a significant improvement in HAM-D response and remission rates, also proving its efficacy and safety. Moreover, some promising results have been described in patients supplemented with SAMe and the probiotic bacteria *Lactobacillus Plantarum* HEAL9, improving mild to moderate symptoms in patients with MDD [141].

In conclusion, SAMe probably exert some benefits in the clinical management of MDD, particularly when combined with traditional and novel therapeutical approaches. However, further studies are needed to clarify the effects of SAMe in depressed patients.

### 4.4. Methylfolate

5-methyltetrahydrofolate (5-MTHF) is the active form of the vitamin B9 (folate). Metabolically, folate cycle is linked to the met cycle and as above-mentioned it takes part in the one-carbon cycle. 5-MTHF is synthesized from 5,10-methylene tetrahydrofolate through the enzyme methylenetetrahydrofolate reductase (MTHFR). 5-MTHF is critical to regenerate met from Hcys, in a reaction mediated by cyanocobalamin (vitamin B12) and the enzyme methionine synthase (MTR) [142]. Thus, 5-MTHF is essential to restore SAMe levels, while stimulating monoamine synthesis mainly through the stabilization and increased production of BH4 [143]. In addition, 5-MTHF may be useful for ameliorating the neuroinflammatory damage in patients with MDD, either as direct and indirect mechanisms [144]. Importantly, patients with MDD show lower serum levels and dietary intake of folate [145]. Furthermore, a genetic polymorphism in the MTHFR gene, the MTHFR C677T variant appears to be associated with an increased risk of suffering from MDD and other psychiatric disorders [146]. Therefore, supplementation with folate and its different forms like 5-MTHF has proven its effectiveness and safety in the treatment of patients with MDD [147]. However, compelling evidence has reported multiple advantages from 5-MTHF in comparison with folate supplementation. For example, 5-MTHF is well-absorbed even under unfavorable gastrointestinal pH, with a great bioavailability, independently from any metabolic defects, also preventing potential negative effects from unconverted folic acid in the peripheral circulation [148]. In addition, supplementation with L-methylfolate may be especially useful for patients with a deficient MTHFR activity, as this enzymatic dysfunction is related with a reduced formation of methylfolate [149]. Conversely, supplementation with 5 mg of folic acid did not report any favorable effect in a 12-week intervention of patients with moderate and severe MDD [150], thereby supporting the notion that methylfolate is the most appropriate supplement related to folate dysfunction.

Current studies support the possible use of 5-MTHF in the clinical management of mental disorders like MDD, prominently as an adjunctive therapy in combination with antidepressants [151]. Papakostas et al. [152] conducted a randomized multicenter clinical trial in patients with partial or no response to SSRIs. They observed that adjunctive L-methylfolate at 15 mg/day constituted an effective, safe and well-tolerated strategy in those patients, although they did not obtain any improvements at lower doses (7.5 mg/day). Interestingly, patients with biomarkers of inflammation, metabolic disorders or folate metabolism-related genetic polymorphisms (or ≥2 of these factors), had the best responses to the adjunctive use of 5-MTHFR [153]. In this sense, it seems that a high BMI > 30, certain inflammatory markers (IL-6, IL-8, CRP), TNF-α and leptin levels were useful markers to predict an effective response to 5-MTHF [154]. In a 12-month, open-label study completed in 68 patients, 38% achieved full recovery without MDD recurrence, and among patients recruited with MDD in remission, 91% were full recovered without recurrence [155]. The use of methylfolate could also be relevant in some vulnerable population like pregnant women. Freeman et al. [156] tested a prenatal supplement, EnBrace HR containing methylfolate in a group of women with history of MDD who were planning pregnancy or pregnant. They observed that women with no active depression experienced a lower rate of depressive relapse, whereas those with active depression reported significant improvements in their depression rating scale. Despite the promising results obtained, 1 participant out of 16 was hospitalized by depression, and further studies are required in this group. Dartois et al. [157] conducted a case series study in 10 treatment-resistant adolescents (80% were women predominantly with a deficient activity of the MTHFR enzyme), showing significant improvements in depression, anxiety, and irritability.

Overall, the use of methylfolate as nutraceutical in MDD have obtained some promising results, while demonstrating its safety and tolerance. However, a recent case report has found a 61-years old woman in remission of psoriasis after the therapy of 15 mg/day of 5-MTHF due to MDD [158]. Further studies are needed in order to identify those groups who may be the most benefited from the use of this compound.

### 4.5. Creatine and Aminoacids

Creatine is an organic compound naturally synthesized in our cells, also found exogenously in different foods, such as fish and meats. Creatine is a well-known supplement among athletes, as this component exerts a plethora of ergogenic benefits. However, recent studies have elucidated the central role of this component either in health and disease conditions as creatine has multitude of cellular and molecular targets [159]. Among some of the most important effects of creatine in the organism highlight its metabolic regulation, with reduced ROS production, a potential anti-inflammatory action and diminished HCys levels [160]. Creatine is synthesized in the liver, kidney and pancreas from glycine, met and arginine. In turn, creatine is a central molecule involved in the aminoacid metabolism, prominently on met cycle, where its synthesis account for approximately 40% of all of the labile methyl groups provided by SAMe as well as an appreciable burden of the Arginine metabolism [161]. Creatine enters in the cell through a Na^+^/Cl^−^ dependent Cr transporter. Then, it is transformed by the enzyme creatine kinase into phosphocreatine, playing a vital role in the cellular bioenergetics, providing an evolutionary advantage for rapid, local and temporal support of energy and mechanical processes [162]. In the brain, creatine supplementation is associated with substantial benefits in the cognitive processing, especially after creatine deficits induced by acute stressors (e.g., exercise, sleep deprivation) or under chronic pathological conditions [163]. Creatine is specifically located in brain regions with high activity, including the hippocampus, choroid plexus cerebellum, cerebral cortex, pontine reticular formation and red nucleus [164]. Patients with MDD appears to present a reduced intake of dietary creatine [165] as well as an altered levels of creatine and an abnormal metabolism of this component in the brain [166]. Because of that, creatine supplementation is being considered as a potential therapeutic approach of MDD.

The molecular basis by which creatine exert its possible antidepressant effects in the brain has been studied in preclinical and animal studies. Creatine acts in the hippocampus and other brain regions modulating the action of some critical targets. For instance, creatine supplementation appears to be sufficient to inhibit the corticosterone-induced decrease of BDNF levels, also activating PI3K/Akt pathway and regulating proteins involved in the synaptic plasticity such as PSD95 [166]. Furthermore, creatine detection in the cerebral spinal fluid (CSF) appears to correlate with serotonin and dopamine levels, suggesting that a proper functioning of these neurotransmitters might be dependent on creatine [167]. Thus, the pro-energetic activity of creatine is critical to favor proper brain functioning. In clinical trials, creatine supplementation (between 2 to 6 g per day) appears to be well-tolerated by patients, with reported antidepressant activity, although it could have significant adverse effects in patients with bipolar depression, who may present an increased risk of developing hypomania/mania [168]. Many of these trials have been conducted in women, as prior in vivo studies demonstrated a greater response of females to creatine, probably due to differences in the creatine metabolism and hormonal milieu [169]. However, further research is required for a better comprehension of the role of creatine in the clinical management of MDD.

In patients with MDD, diminished levels of some aminoacids have been detected in their metabolic profile, including met, phenylalanine, tyrosine and tryptophan [170]. Thus, the use of aminoacids as nutraceutical has provided certain benefits in some studies with MDD. Phenylalanine and, more prominently, tyrosine both appear to be implicated in the synthesis of dopamine and norepinephrine [171], also influencing the neuroinflammatory response in the brain of patients with MDD [172,173]. Regarding phenylalanine, previous works developed some decades ago started to investigate the role of this aminoacid in MDD [174,175]. However, little evidence supports the use of this supplement in depressed patients and some studies have found potential harmful effects in depressed patients with Parkinson disease [176,177] and during pregnancy [178]. Tyrosine also has controversial evidence. Gelenberg et al. [179] completed a trial in 65 people with depression received either 100 mg/kg of tyrosine, 2.5 mg/kg of a common antidepressant or a placebo each day for four weeks. However, they concluded that the antidepressant efficacy of tyrosine could not be demonstrated. On the other hand, patients with low levels of dopamine may benefit from the supplementation of tyrosine, as shown by Mouret et al. [180]. Further studies are needed to define a possible role of tyrosine in MDD, as the available data are not enough to describe whether this supplement is of aid or not [181]. Tryptophan seems to be a more effective aminoacid in the therapy of MDD, as demonstrated by different clinical trials [182]. A recent systematic review conducted by Kikuchi et al. [183] showed that taking 0.14–3 g of tryptophan per day, together with a proper diet can improve the mood of healthy individuals. Similar results are obtained in depressed patients, where higher consumption of dietary tryptophan resulted in less depressive symptoms and decreased anxiety [184]. Previous studies have found significant differences in the efficacy of tryptophan supplementation according to the genotype and sex, although many more efforts are needed in this field to predict the recommendation of this nutraceutical [185]. The main reason of the effectiveness of tryptophan in depression is because of its direct effects on serotonin synthesis. However, some of the etiological and pathophysiological mechanisms of MDD like stress, an increased release of pro-inflammatory cytokines or gut dysbiosis might induce the conversion of tryptophan to quinolinic acid, associated with neurotoxic properties [42,186]. Indeed, targeting this metabolic reprogramming might be especially useful for patients with MDD [187]. Thus, tryptophan might be a supplement of great aid specially in combination with other approaches directed to low the neuroinflammatory reaction, gut dysbiosis and/or stress circuit.

### 4.6. Prebiotics and Probiotics

Probiotics consist of live bacteria present in certain foods (yogurt, kefir, tempeh, etc.) or supplements, whereas prebiotics are complex carbohydrates that only bacteria can digest. The research in this field has prominently increased in the last decades, concretely finding the nutritional modulation of microbiota for prevention and treatment of neuroimmune and neuroinflammatory diseases [188]. In the tangled mess of the multiple factors involved in MDD, gut dysbiosis and intestinal complaints are frequent. Evidence alleges that probiotics are effective in ameliorating depressive symptoms when administered with antidepressants, not only for the gut–brain axis but also for the metabolic disturbances of comorbidities that are particularly frequent in MDD (metabolic syndrome) [189]. Nevertheless, standardized methods to measure probiotics efficacy in clinical trials remain unclear.

That the microbiome influences thinking, feeling and acting has been identified recently. The group of microorganisms that are able to modulate immune, endocrine and metabolic host signaling affecting neuroinflammation and neurotransmission are also denominated “psychobiome” [190]. Some species like (*Roseburia intestinalis*, *Eubacterium spp*., *Bacteroides spp*. and *Akkermansia muciniphila*) have been isolated from human gut microbiota and cultivated. These species provide properties such as butyrate, propionate and more bioactive production, that other common probiotics like bifidobacterial and lactobacilli mostly found in fermented food do not confer [191]. The interest of *Akkermansia muciniphila* resides in its modulation of host serotonin system. This mechanism is well studied in animal models where it was observed that bacterial extracellular vesicles have significant effects on mRNA expression of genes in colon and hippocampus from serotonin signaling pathway [192]. Metagenomic sequencing confirms reductions in microbial diversity and relative abundance of several genera like *Akkermansia spp.* in patients with anxiety- and depressive-like symptoms [193]. In some animal models of obesity and mental disorders, supplementation with a subtype of *Akkermansia muciniphila* denoted positive outcomes ameliorating spatial memory, increasing Nissl bodies in neurons of hippocampus, and increasing relative fecal abundance of *Bifidobacterium* [194].

One suggestion for a better management of microbiota targeting in MDD is identifying metaorganism biomarkers that make the difference with respect to healthy reference subjects. These biomarkers are microbiome taxa clustering and neurocircuit-relevant metabolic networks (GABA, butyrate, monoamines, etc.) [195]. A meta-analysis about the effects of probiotics on depression demonstrated there is significant reduction in the depression scale score in the population aged under 60, but not in people aged over 65 [196]. In this sense, elder individuals with chronic diseases are less effective to respond to any treatment and they would need more time to see significant results. In other trial with 83 participants (mean age 43.9 years), significant benefits were observed ameliorating anxiety and depressive symptoms when administering a multispecies probiotics product [197].

Conversely, prebiotics, which are the nutrients that only microbiota degrade to obtain bioactive compounds, are preferably advised in combination with probiotics. Prebiotics alone have provided controversial data, with mild or no evidence of significant improvements in patients with MDD [198]. Fructo-oligosaccharides and galacto-oligosaccharides are every time more frequently found in fortified foods, now that they appear naturally in low qualities and seem to be of interest for human health [199]. In any case, probiotics and prebiotics offer better benefits when combined, but further research is still required to demonstrate the clinical significance in MDD [200].

### 4.7. Micronutrients

As above-mentioned, vitamin D is the nutraceutical with the greatest scientific evidence supporting its use in patients with MDD. However, there are further studies investigating the role of other vitamins in depressed individuals as well as its potential use in the clinical management of these patients. Not only folate and its derivates but also other vitamins from the B group appear to play a central role in the pathogenesis of MDD. Particularly, lower levels of vitamin B1 (thiamine), B2 (riboflavin), B3 (niacin), B6 (pyridoxine) and B12 are reported in patients with MDD [201]. Therefore, some studies have reported certain benefits from supplementing with vitamin B complex on modo and quality of life in patients with major depression [202]. Nonetheless, a recent meta-analysis and systematic review developed by Young et al. [203] concluded that despite vitamin B supplementation could be effective for healthy individuals and at-risk populations for stress, there was no real effect of vitamin B complex in the improvement of depressive symptoms. These results were supported by Markun et al. [204] who did not extract any significance of the supplementation of B12 in depressive symptoms neither alone nor in combination with vitamin B complex. This could be due to the gut dysbiosis found in patients with MDD, as many bacteria are involved in the regulation of some critical vitamins’ levels in the organism [205]. Thus, the use of probiotics might be of aid to improve the levels of vitamins in the serum while ameliorating the levels of negative circulating parameters in people with any disease established [206]. In patients with MDD, it could be not as simple. For instance, Reininghaus et al. [207] used supplementary probiotic treatment with and without vitamin B8 (biotin) for 4 weeks in 82 depressed patients. They show that the use of probiotic alone despite improving the microbial profile did not exert any clinical benefit in comparison to placebo. However, when combined with B8 significant improvements were observed in the clinical management of those patients. This is an example that a combination of certain nutraceuticals could be more effective in the therapy of such a complex disease, always in a context of a proper diet and lifestyle. Other vitamins like vitamin A (retinol) and vitamin E (tocopherol) are also reduced in the serum of patients with MDD [208]. Therefore, there are some clinical and preclinical studies describing a favorable antidepressant action of pro-vitamin A (beta-carotene) and vitamin E, mainly by its antioxidant and anti-inflammatory effects [209,210]. However, there is still little evidence supporting its use as supplements. Currently, it seems more appropriated to consider the inclusion of these vitamins contained in food. A systematic review done by LaChance and Ramsey [211] claimed that the highest scoring animal foods were bivalves, such as oysters and mussels; various seafoods; and organ meats, whereas the highest scoring plant foods were leafy greens, lettuces, peppers and cruciferous vegetables. These foods are specially rich in minerals and trace elements like iron, zinc, magnesium, potassium, selenium, omega-3 fatty acids (EPA and DHA) and vitamins A, B1, B6, B9, B12 and C.

Together with vitamins, minerals are also vital micronutrients for a proper functioning of the brain [212]. Minerals could be divided in macrominerals (calcium, magnesium, chloride, phosphorus, sodium, potassium and sulfur) and trace elements (iron, iodine, copper, manganese, cobalt, zinc, fluoride and selenium). In general, macrominerals are required in higher doses (>100 mg/day) than trace elements (1–100 mg/day) [213]. Altered levels of either macrominerals and trace elements have been considered as important contributing factors in the pathophysiology of MDD. In particular, reduced levels of calcium, magnesium, iron, manganese, selenium and zinc are observed in depressed patients, with augmented detection of serum copper [214]. Calcium is an essential ion implicated in the regulation of different physiological processes in the brain, including neuronal gene expression, energy production, membrane excitability, synaptogenesis, synaptic transmission and other superior functions underlying learning, memory and cell survival [215] Because of that, one study has described specific benefits from calcium supplementation, particularly when combined with vitamin D [216]. However, due to the greatest efficacy of Vitamin D, the role of this nutraceutical has been little studied. Magnesium is another macromineral involved in the regulation of complex cognitive processes as it has been widely demonstrated in previous animal models [217]. Because of that, magnesium has been proposed as a potential antidepressant nutraceutical for almost 100 years ago and numerous preclinical and clinical studies currently support its use [218]. Rajizadeh et al. [219] evaluated the role of magnesium supplementation on patients with MDD with magnesium deficiency in the serum. They reported significant benefits from daily consumption of 500 mg magnesium oxide per day on depression status and hypomagnesemia. In accordance with these results, Tarleton et al. [220] conducted a 6-weeks intervention trial with magnesium in comparison to 6 weeks without any supplement in patients with mild and moderate MDD. They obtained favorable outcomes in those individuals regardless of age, gender, baseline severity of depression, baseline magnesium level, or use of antidepressant treatments. A potential advantage of the use of magnesium resides on its rapid action, as previous studies have demonstrated the quick recovery from patients supplemented with magnesium (1–2 weeks) [220,221]. However, recent systematic reviews demonstrated that despite the fact that magnesium may be favorable in the clinical management of MDD, the level of evidence supporting its use is insufficient, and future clinical trials should be directed to evaluate the action of magnesium alone or in combination with antidepressants, in order to maximize its benefits [222]. On the other hand, there are more robust data encouraging the use of zinc supplementation in patients with MDD, as it seems to augment the efficacy of antidepressants [223] even in non-responsive patients [224]. Notwithstanding there is still little and inconsistent evidence regarding selenium and manganese supplementation, further studies are needed in this field, as they also control some central processes and functions in the brain [225,226]. In case of iron, it is well-known its central role in brain organization. Patients with iron deficiency present poor brain myelination, impaired monoamine, GABA and glutamate neurotransmission and augmented oxidative stress in the brain tissue [227]. Thus, previous studies have demonstrated the relevance of iron supplementation in patients with iron deficiency anemia, as it appears to be associated with a reduced risk of suffering from MDD in those individuals [228]. However, we have not noticed about any study regarding the nutraceutical use of iron in MDD. On the other hand, the benefits from supplementation with trace elements appears to work through the modulation of critical events involved in the pathophysiology of MDD, targeting oxidative stress, monoaminergic system, systemic and local inflammation, GABAergic system, sleep regulation and neuroprotective effects mediated by BDNF [229]. Ultra-trace minerals are defined with less than 1 microgram/day required. Here, it could be included some minerals like aluminum, vanadium, boron and nickel. Little data are collected about the role of these elements in MDD, except for lithium. Lithium is another ultra-trace element with reported efficacy in the treatment of bipolar disorders. Indeed, it is considered the first choice in patients with this condition [230]. In patients with unipolar MDD, the use of lithium is less extended. Nevertheless, there are some studies recommending the use of this ultra-trace element, particularly in the long-term prophylaxis for non-responder patients, as well as to prevent suicidal thoughts [231,232].

### 4.8. Plant Derived Bioactive Compounds

Currently, there is no consistent and unified definition of bioactive compounds in the available literature. However, an approximated description of this term could be “compounds which have the capability and the ability to interact with one or more component (s) of living tissues by presenting a wide range of probable effects” [233]. Thus, a growing interest has been placed in the beneficial role of different bioactive compounds either in health maintenance or to prevent and aid in the clinical management of multiple diseases [234]. In general, bioactive compounds are found in small quantities in different foods, generally vegetables, fruits and plant-based products (mainly polyphenols, alkaloids, terpenes or saponins) [235]. However, animal foods, fungi and even marine microbials might provide interesting bioactive compounds that may be potentially used as nutraceuticals [236,237,238]. Therefore, there are many bioactive compounds provided by different types of food which may be critical for a proper functioning of the brain, mainly due to their anti-inflammatory and antioxidant properties [239]. In this section we will only summarize some of the most important bioactive compounds derived from plants and their potential applications in MDD.

One of the most relevant bioactive compounds with proven neuroprotective effects are contained in the coffee. Caffeine is perhaps the best-known component, although there are many other components including polyphenols, especially chlorogenic acids (in green beans) and caffeic acid (in roasted coffee beans), alkaloids (caffeine and trigonelline) and the diterpenes (cafestol and kahweol) [240]. Overall, these bioactive compounds interact together to exert their beneficial effects in the brain. However, here we will only focus on caffeine, as it is the most widely nutraceutical used derived from the coffee. Either coffee or caffeine consumption has been associated with decreased risk of depression [241,242]. In addition, there are some studies supporting the promising role of caffeine in many depressive symptoms, enhancing the efficacy of antidepressant therapy [243]. This effect is due to the non-selective antagonist of caffeine for adenosine receptors A1/A2, leading to significant improvements in the treatment of motivational dysfunction in patients with MDD, acting through increase levels of the neurotransmitter dopamine [244]. However, the benefits from caffeine in patients with MDD appears to be dose dependent, as high doses of this compound may result in thymic dysregulation, favor mixed affective states and disturbances in the circadian profiles and worsening of anxiety symptoms [245]. Another alkaloid with growing interest is theobromine, which is abundantly contained in cocoa, in combination with flavonoids and caffeine. Interestingly, previous studies appear to indicate a possible role of theobromine as a cognitive modulator, although the role of this component is underexplored [246]. Required doses of this nutraceutical should not be more than 250 mg, as occurring with caffeine, where higher doses of this compound may provide negative effects [247]. This could be a potential line of future research in the field of MDD and other mental disorders, as theobromine seems to be associated with less undesired effects than caffeine and future studies are required [248].

Polyphenols are a great group of bioactive compounds synthesized exclusively by plants presenting chemical features related to phenolic substances, providing strong antioxidants properties [249]. In a simple manner, polyphenols are classified in flavonoids and non-flavonoids. The first are categorized in six subgroups: anthocyanins, chalcones, flavanones, flavones, flavanols and isoflavonoids [250]. An important flavonoid is quercetin. Flavonoids are important molecules with tested antidepressant properties. Animal models have demonstrated noticeable effects from oral administration of multiple flavonoids in the pathophysiology of MDD including an improved functioning of the monoaminergic system, GABAergic transmission, BDNF activity and amelioration of the neuroinflammatory response in the brain [251]. Some of the most used antidepressant flavonoids in preclinical studies are summarized by German-Ponciano et al. [252]. However, to our knowledge there are no studies evaluating the efficacy of flavonoids supplementation in patients with MDD. Further studies should be directed to evaluate the role of this compounds in addition with antidepressant therapies, always accompanied with a diet rich in vegetables and fruits, where the flavonoids are present in high concentrations. Among nonflavonoids there are some studies evaluating the role of curcumin, a phenolic compound present in *Curcuma longa*. Some of the proposed mechanisms from this polyphenol include improvements in BDNF activity, serotoninergic and dopamine transmission, apart from the antioxidant and anti-inflammatory properties [253]. A recent meta-analysis suggested that combined use of curcumin and antidepressants might be effective in the clinical management of depressive and anxiety symptoms in patients with MDD [254]. However, the authors concluded that given the low sample size, additional studies are required in this area, prominently in western countries, as many of these studies were conducted in Asian countries. Hydroxytyrosol, another phenolic compound found at high doses in olive oil, has also reported substantial benefits in the management of depressive behaviors in mice models [255] and given the multiple benefits of this component in mental health and disease [256,257], we encourage for further research in this area. More evidence supports the use of resveratrol in animal models of MDD, acting mainly by the following mechanisms: regulation of HPA axis, increased BDNF activity, neurogenesis and monoamines production and decrease neuroinflammation, mitochondrial damage and oxidative stress [258,259]. However, similar to some of the previous bioactive compounds, the clinical application of resveratrol is quite limited and future studies will be critical to understand and prove the role of resveratrol in the clinical management of MDD.

Finally, we would like to highlight the possible role of cannabidiol (CBD), a natural compound obtained from *Cannabis sativa*. Although some authorities do not consider CBD as a nutraceutical, other authors argue that CBD is, indeed, either a nutraceutical and drug with multiple effects in the brain and multiple tissues in the body [260]. At some extent, the cause of this controversy resides on the social view of the products derived from *Cannabis sativa*, specially tetrahydrocannabinol (THC). Although this molecule mostly exerts psychoactive and negative effects, CBD has totally different actions and in many cases opposite effects, not inducing euphoria but providing antipsychotic, anxiolytic, antidepressant, antiepileptic and anti-inflammatory properties [261]. The effects of CBD in MDD are numerous. On the one hand, it interacts with serotoninergic, glutamatergic and GABAergic transmission, as well as with the endocannabinoid system [262]. In addition, CBD is responsible for multiple cellular and molecular changes in brain regions related to MDD neurobiology, increasing the levels of BDNF, neurogenesis and neuroplasticity in these areas [263]. Despite all the benefits and described mechanisms of CBD in MDD, there are no clinical studies conducted with the use of this nutraceutical, although some promising results are being obtained in other psychiatric disorders like schizophrenia and anxiety [264]. Additional studies implementing CBD in depressed patients might represent a potential approach in the clinical management of MDD.

## 5. Conclusions and Future Directions

Multiple animal studies have proven the relevance of nutraceuticals as effective antidepressant agents, targeting many of the pathophysiological mechanisms occurred in MDD. However, the clinical evidence of their use is limited. In Table 1, main nutraceuticals evaluated in MDD are summarized. Omega 3 fatty acids, vitamin D, SAMe and methylfolate are the nutraceuticals with most effects proven. Micronutrients, prebiotics, probiotics, carnitine, aminoacids and plant-derived bioactive compounds are showing some promising results, but mainly in animal models. This is in part surprising due to the growing interest and economic sources placed on many nutraceuticals, although it is also true that this field of research is still in its infancy. Thus, finding an appropriate context on the use of nutraceuticals in MDD is warranted.

It is important to understand that nutraceuticals rather than inhibit a specific molecular pathway as a pharmacological agent may do, is directed to broader molecular and cellular and targets and as the proverb states better not to “bite off more than you can chew”. In other words, nutraceuticals could be a potential support and adjunctive therapy in patients with MDD, but always combined with accepted therapies, as the benefits then could be greater. In addition, it would be advisable to develop further strategies evaluating the best dosage form of nutraceuticals in patients with MDD. A recent work conducted by Sarris et al. [265] studied the effect of a combination of different nutraceuticals previously mentioned (SAMe, omega 3, folinic acid, tryptophan, zinc and other cofactors) in patients with MDD, contained in two pills and two tablets given to the individuals twice per day for 8 weeks, They observed that the effect of placebo was superior to that combination of nutraceuticals. However, there were some plausible explanations to these results, including high expectancies that make people more prone to suffer from placebo effect, together with possible and unknown interactions of these nutraceuticals in terms of pharmacokinetics and pharmacodynamics. In this sense, we recommend not to focus on the combination of various isolated nutraceuticals components, as we also have foods that contain an adequate nutraceutical mixture integrated in their “matrix food”, as the therapeutic success of a food and its nutraceutical components is much more than the sum of the parts, but as a whole [266]. The important antidepressant properties of some nutraceutical supplements make them worth to conduct further efforts and research. However, we must not forget that nutraceuticals contained in a proper food matrix are generally more effective than those given as free supplements [267]. Thus, an integrative perspective is required here, providing personalized strategies for each individual. An interesting approach for future studies would be the combination of supplements (particularly, in those patients with reduced serum level of a certain nutrient or certain genetic polymorphisms) and the inclusion of some foods and dietary patterns with high content in that nutraceutical, aiding to maximize the bioavailability and benefits of this component. The release of nutraceuticals from food matrix or nanocarriers in gastrointestinal fluids, their solubilization, their interaction with other components of gastrointestinal fluids, their absorption by the epithelial layer, and the chemical and biochemical transformations in the epithelial cells are critical to augment the bioavailability and action of nutraceuticals [268]. Future studies on this field will bring more benefits in the clinical and translational use of nutraceuticals in many conditions, including MDD. Not less important to mention is that self-medication is risky. Some supplements are indeed components that our own body need to synthesize in normal conditions and the uncontrolled uptake make some people become dependent on these. In this review, our aim was also to provide knowledge of certain components that are naturally present in many foods, and note that, when referring to certain nutraceuticals that can be taken by pills of supplements, the accompanying specialist advice must be always present.

**Figure 1:**
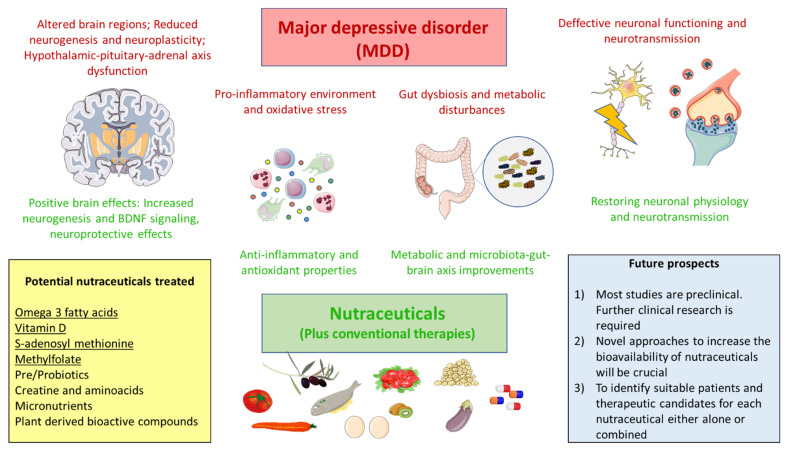
A Graphic summary of the ideas transmitted.

In this review, we summarize some of the most relevant and updated researches regarding the use of nutraceuticals in preclinical and clinical studies of MDD. In Figure 1, main applications and biological targets of nutraceuticals in MDD are represented. Our work aims to provide a comprehensive and detailed information either for scientific and general public, aiding to identify potential and useful applications of nutraceuticals in MDD while encouraging for future efforts in this promising field. The brain is an extraordinarily complex organ which is dependent from multiple biological and non-biological factors. All these variables are equally important to promote an adequate functioning of the brain. Therefore, it should not be forgotten that apart from the use of nutraceuticals and other medical interventions, a proper physical activity, diet, rest and other lifestyle and psychosocial interventions are also critical approaches in the clinical management of patients with MDD.

## Figures and Tables

**Table 1:** A general summary of the main nutraceuticals, dietary sources and their preclinical/clinical evidence.

Nutraceutical	Main Dietary Sources	Probable Antidepressant Effects	Clinical Evidence	Side Effects or Limitations	References
Omega 3	Oily fish, nuts, seeds	Targeting of lipid rafts and G coupled protein receptors; Influencing neurotransmission; Stimulation of myelin proteins; Improved cognitive functioning and neuronal cytoarchitecture; Positive influence in the endocannabinoid system and BDNF activity; Anti-inflammatory effects	Major clinical efficacy appears at combined doses of EPA (>60%) + DHA	Long-term results; More effective in patients with mild to moderate depressive symptoms	[81,82,83,99,100]
Vitamin D	Oily fish, dairy products, eggs, seafood	Immune and microbial homeostasis; Serotonin synthesis; Circadian clock; Increased BDNF activity	Vitamin D_3_ at a dose of 50,000 IU/week show possible antidepressant effects also improving sleep quality vitamin D deficiency as a possible risk factor for late-life depression	Further clinical evidence is warranted	[114,115,116,118,120]
SAMe	Endogenously synthesized or supplements	Influence monoamine synthesis and activity; Improved methylation status and BDNF activity; Anti-inflammatory effects and targeting microbiota–gut–brain axis.	Combination of SAMe with standard antidepressants but not alone have demonstrated the safety, efficacy and tolerability of this component. One study also shows improved action when combined with probiotics	Possible sex-dependent effects;	[129,130,131,132,133,134,135,136,137,138]
Methylfolate	Endogenously synthesized or supplements	Improved monoamine synthesis and activity; Anti-inflammatory effects; Restoring SAMe levels in the organism	More benefits than supplementation with folate are obtained; 15 mg of methylfolate but not 7.5 exert possible antidepressant effects with standard therapies; High BMI, inflammatory mediators and leptin levels appears to act as predictive; Pregnant women or individuals with reduced MTHFR activity may be potential candidates for this nutraceutical	One case report study described a relapse of psoriasis in a 61 years old woman after 15 mg/day of methylfolate; Further evidence is warranted	[149,150,151,152,153,154]
Prebiotics	Fruits, vegetables, whole grains, legumes	Growing of beneficial bacteria	Some studies have found mild efficacy of prebiotic in MDD, but more prominently with probiotics	Prebiotics alone may not have any positive action for patients with MDD	[195,196,197]
Probiotics	Yogurt, kefir, kombucha, tempeh, miso	Growing of beneficial bacteria in the gut	Certain probiotic species alone or in combination improves clinical parameters in patients <65 years	Elderly people appear to be less sensitive to probiotics	[190,191,192,193,194]
Carnitine	Fish, meats, dairy products	Anti-inflammatory effects; Antioxidant; Improved metabolic profile; Cognitive enhancement; Neuroplastic effects; Increased BDNF activity; Probable effects in neurotransmitter functioning	2 to 6 g per day of creatine supplementation appears to be well-tolerated and effective in patients with MDD	In patients with bipolar depression, it may increase the risk of suffering from hypomania/mania; Most studies are conducted in women (Sex-dependent effect); Further evidence is warranted	[160,161,162,163]
Tyrosine,	Poultry, dairy products, avocado, nuts, pumpkin/sesame seeds	Involved in dopamine and norepinephrine synthesis; Anti-inflammatory effects	Combined use of 100 mg/kg tyrosine plus imipramine show no conclusive antidepressant activity in comparison to placebo; Patients with low levels of dopamine may beneficiate from this nutraceutical	Available data is still controversial	[176,177,178]
Phenylalanine	Red meats, fish, eggs, dairy products, soy, nuts.	Involved in dopamine and norepinephrine synthesis; Anti-inflammatory effects	Some clinical studies described some favorable effects of phenylalanine in MDD	There are no recent studies conducted in the use of this nutraceutical; it may be related to important adverse effects in patients with Parkinson Disease and pregnant women	[171,172,173,174,175]
Tryptophan	Soy, fish, poultry, eggs, dairy products, cocoa	Involved in serotonin synthesis	0.14–3 g of tryptophan per day in a context of a healthy diet may favorably influence patient’s mood	Tryptophan may be converted to quinolinic acid in patients with MDD (Neurotoxicity)	[179,180,181,182,183]
Vitamin B	Whole grains, meats, eggs, dairy products, seeds, nuts, dark leafy vegetables, fruits	Anti-inflammatory and antioxidant properties	Most trials are negative. However, dual supplementation of probiotics plus vitamin B8 obtained some clinical improvements	Including a varied diet with high vitamin content is much more effective than supplementation according to available scientific data	[198,199,200,201,204]
Vitamin A	Fruits and vegetables, meats, fish and dairy products		Serum levels of this component are reduced in patients with MDD.		[205,206,207]
Vitamin E	Dark leafy vegetables, nuts, seeds, vegetable oils		Serum levels of this component are reduced in patients with MDD		[205,206,207]
Calcium	Dairy products, fish, dark leafy vegetables	Neuronal gene expression, energy production, membrane excitability, synaptogenesis, synaptic transmission and cognitive functions	One study obtained favorable results from calcium plus vitamin D supplementation, but not alone	This nutraceutical has not provided too much interest	[212,213]
Magnesium	Dairy products, fish, dark leafy vegetables, legumes, nuts, seeds	Involved in complex cognitive processes	Daily consumption of 500 mg magnesium oxide per day improved depression status and hypomagnesemia; A 6-weeks intervention trial with magnesium in comparison to 6 weeks without any supplement in patients with mild and moderate MDD, regardless of age, gender, baseline severity of depression, baseline magnesium level, or use of antidepressant treatments; Magnesium exerts rapid actions (1–2 weeks)	Little evidence available	[216,217,218]
Zinc	Dairy products, fish, dark leafy vegetables, legumes, nuts, seeds, fish, red meat, poultry.	Pleiotropic effects	Zinc combined with antidepressants maximize clinical outcomes even in non-responsive patients		[220,221]
Trace elements (Iron, selenium, manganese)	Meat, fish, cereals, milk and dairy foods, vegetables and nuts	Targeting oxidative stress, monoaminergic system, systemic and local inflammation, GABAergic system, sleep regulation and neuroprotective effects mediated by BDNF	Iron supplementation might provide prophylactic effects in patients with anemia	Further evidence is warranted	[222,223,224,225,226]
Ultra-trace elements (Lithium)	Potato, vegetables, fish and seafood	Involved in complex cognitive processes	Some studies reported positive outcomes from long-term prophylaxis for non-responder patients, as well as in the prevention of suicidal thoughts	Most studies are conducted in patients with bipolar disorder	[227,228,229]
Alkaloids (Caffeine and theobromine)	Coffee, cocoa	Pleiotropic effects (Non selective antagonist of adenosine receptors)	Different studies have demonstrated the antidepressant effects of caffeine and probably of theobromine	High doses of both components are associated with increased anxiety, depressive and negative symptoms	[238,239,240,241,242,243,244,245]
Flavonoids polyphenols	Vegetables and fruits	Improved functioning of the monoaminergic system, GABAergic transmission, BDNF activity and amelioration of the neuroinflammatory response in the brain		No clinical studies have been conducted	[248,249]
Nonflavonoids polyphenols	Curcumin (Curcuma), resveratrol (grapes) Hydroxytyrosol (Olive oil)	Improving BDNF activity, serotoninergic and dopamine transmission, antioxidant and anti-inflammatory properties	Some studies have proven antidepressant benefits from curcumin in Asian patients with MDD	Further evidence is warranted	[250,251,252,253,254,255,256]
CBD	Only supplements	Influences serotoninergic, glutamatergic and GABAergic transmission, as well as the endocannabinoid system; Targeting of multiple cellular and molecular components, increasing the levels of BDNF, neurogenesis and neuroplasticity	-	There are no clinical studies conducted probably by its social perception	[257,258,259,260,261]

## Data Availability

Data sharing not applicable.
